# MiR-128 reverses the gefitinib resistance of the lung cancer stem cells by inhibiting the c-met/PI3K/AKT pathway

**DOI:** 10.18632/oncotarget.12283

**Published:** 2016-09-27

**Authors:** Jingjin Jiang, Xiaoning Feng, Wenjing Zhou, Yue Wu, Yunmei Yang

**Affiliations:** ^1^ Department of geriatrics, First Affiliated Hospital, School of Medicine, Zhejiang University, Hangzhou 310003, China; ^2^ Division of Hepatobiliary and Pancreatic Surgery, Department of Surgery, First Affiliated Hospital, School of Medicine, Zhejiang University, Hangzhou 310003, China

**Keywords:** PC9-CSCs, miR-128, gefitinib, c-met, PI3K/AKT

## Abstract

Gefitinib is a first line anti-tumor drug used for the treatment of patients with non-small cell lung cancer (NSCLC) harboring EGFR mutations. However, the drug resistance to gefitinib limits its clinical application. Here, we observed the CSCs of PC9 are obviously resistant to gefitinib compared with the non-CSCs. Furthermore, we found the gefitinib failed to suppress the PI3K/AKT pathway in the PC9-CSCs. Mechanically, we showed significant down-regulation of miR-128 in the PC9-CSCs compared with the non-CSCs. Overexpression of miR-128 significantly increased the sensitivity of PC9-CSCs to gefitinib-induced apoptosis. In addition, the gene of c-met was proved to be directly inhibited by miR-128. Enforced expression of c-met could “rescue” the miR-128 promoted apoptosis and cleavage of caspases in PC9-CSCs treated with gefitinib. Thus, these results indicate that the miR-128/c-met pathway enhances the gefitinib sensitivity of the lung cancer stem cells by suppressing the PI3K/AKT pathway.

## INTRODUCTION

Non-small cell lung cancer (NSCLC) represents as the most common type of lung cancer and the leading cause of cancer-related death around the world [1, 2]. The repeated use of anti-cancer drugs usually induces the acquisition of resistance in NSCLC cells [3]. Recently, studies have demonstrated that the existence of small population of cancer stem cells (CSCs) is an important mechanism for chemoresistance [4].

CSCs own the multilineage differentiation potential and the ability of self-renewal. As the CSCs can differentiate and generate heterogeneous cell populations to constitute the tumor, they are considered as the tumor-initiating cells [5]. CD133 is encoded by the prominin 1 (PROM1) gene in humans [6]. In several tumors including lung cancer, CD133 has been considered as a molecular marker to identify the CSCs, and the CD133 positive lung cancer cells are reported to exhibit the stem-like probabilities of tumorigenicity, proliferation, and self-renewal [7–9]. Increasing evidence reveals that the CD133^+^ populations of CSCs in NSCLC are responsible for the high resistance to chemotherapeutic drugs and tumor relapse [10, 11]. Therefore, killing the should be the strategy to treat the NSCLC more effectively.

Gefitinib, which is one of the epidermal growth factor receptor (EGFR) tyrosine kinase inhibitors (TKIs), is widely used for patients with NSCLC, especially the ones harbor somatic mutations in the EGFR gene [12]. Although the gefitinib now is the first-line agent for the treatment of lung cancer, the acquired resistance to gefitinib becomes the Major obstacle after a period of treatment [13]. PC9 cells which express the EGFR exon 19 deletion mutation and are sensitive to gefitinib treatment [14]. However, as the researches have proved that the CSCs are involved in resistance to gefitinib in NSCLC [15], it's urgent to identify the mechanisms and reverse the resistance of PC9-CSCs to gefitinib.

MicroRNAs (miRNAs) are a family of small, non-coding RNAs, which regulate the expression of targeted genes by binding to the mRNAs at the 3′untranslated region (3′ UTR) [16, 17]. Many studies have showed that miRNAs function as regulators in a number of basic biological processes, such as cell proliferation, differentiation, metabolism and apoptosis. Therefore, the miRNAs play important roles in cancer initiation and development [18, 19]. Moreover, aberrantly expression of miRNAs has also been reported to induce drug-resistance in cancers, because the drug-resistance related genes could be changed by the miRNAs regulation [20]. In the present study, we found that the miR-128 expression was decreased in PC9-CSCs. We demonstrated that overexpression of miR-128 could reverse the gefitinib resistance by inhibiting the PI3K/AKT pathway.

## RESULTS

### The lung cancer stem cells are resistant to gefitinib compared with the non-CSCs

**Figure 1:**
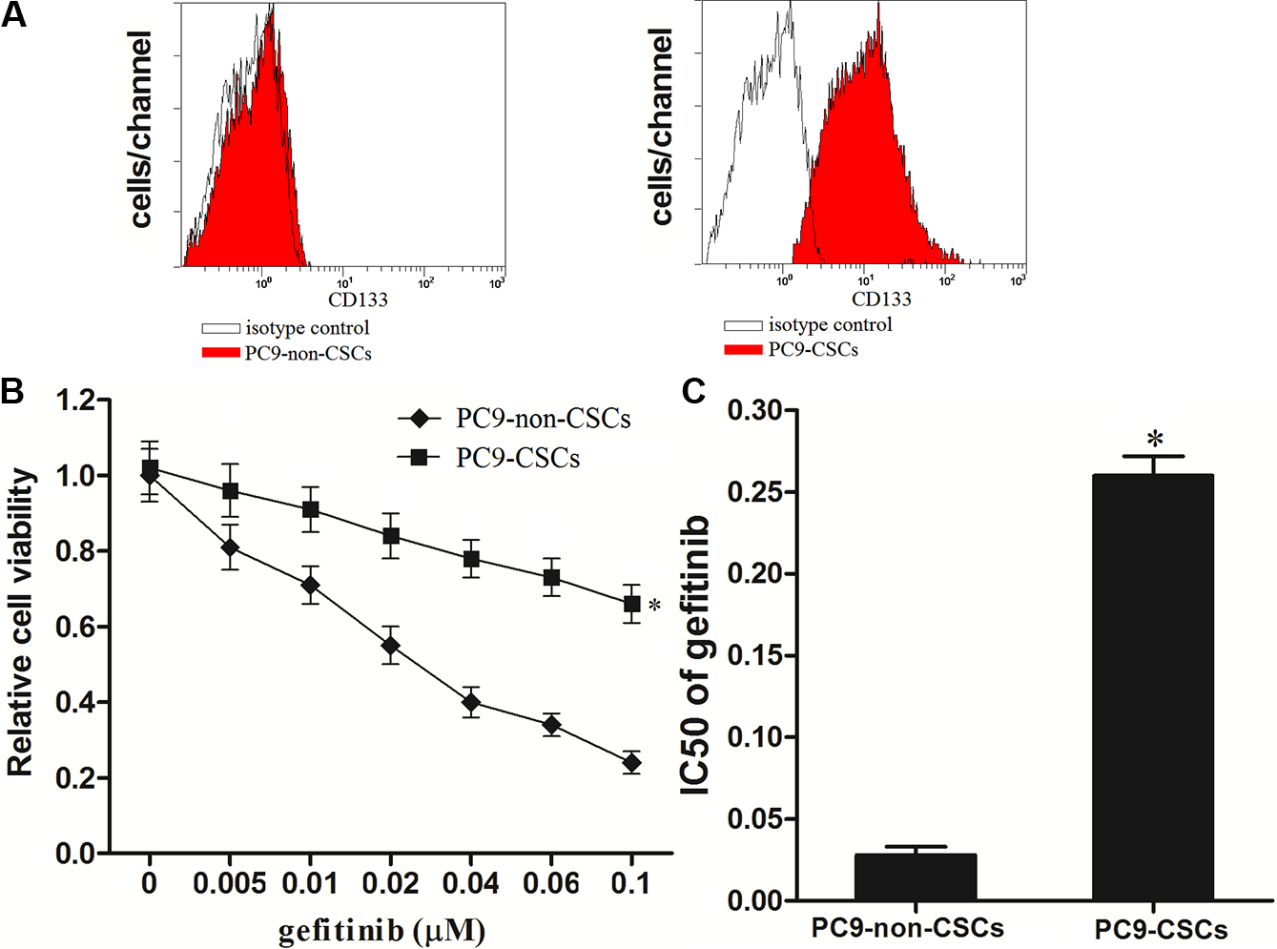
Lung cancer stem cells are resistant to gefitinib (**A**) The PC9-CSCs population was sorted as CD133^+^ cells, and the CD133^−/low^ cells were sorted as the PC9-non-CSCs on the flow cytometry. (**B**) The sensitivity of PC9-CSCs and PC9-non-CSCs was evaluated by MTT assays. (**C**) The IC50 of PC9-CSCs and PC9-non-CSCs to gefitinib was calculated according to the viability curves performed by MTT assays. *P < 0.05.

To study the gefitinib resistance in lung cancer, we separated the CSCs and non-CSCs from the PC9 cell line. The PC9-CSCs was isolated by using the CD133 antibody, and the efficiency of separation is shown in Figure 1A, We observed that although the PC9-non-CSCs are sensitivity to gefitinib-induced cell death, the PC9-CSCs showed obvious resistance to gefitinib (Figure 1B). Intuitively, the IC50 of gefitinib to PC9-CSCs is 9.29 fold higher than the PC9-non-CSCs (Figure 1C). These results indicate that the CSCs of lung cancer show resistance to gefitinib.

### PC9-CSCs are resistant to the gefitinib-induced inactivation of PI3K/AKT pathway

**Figure 2:**
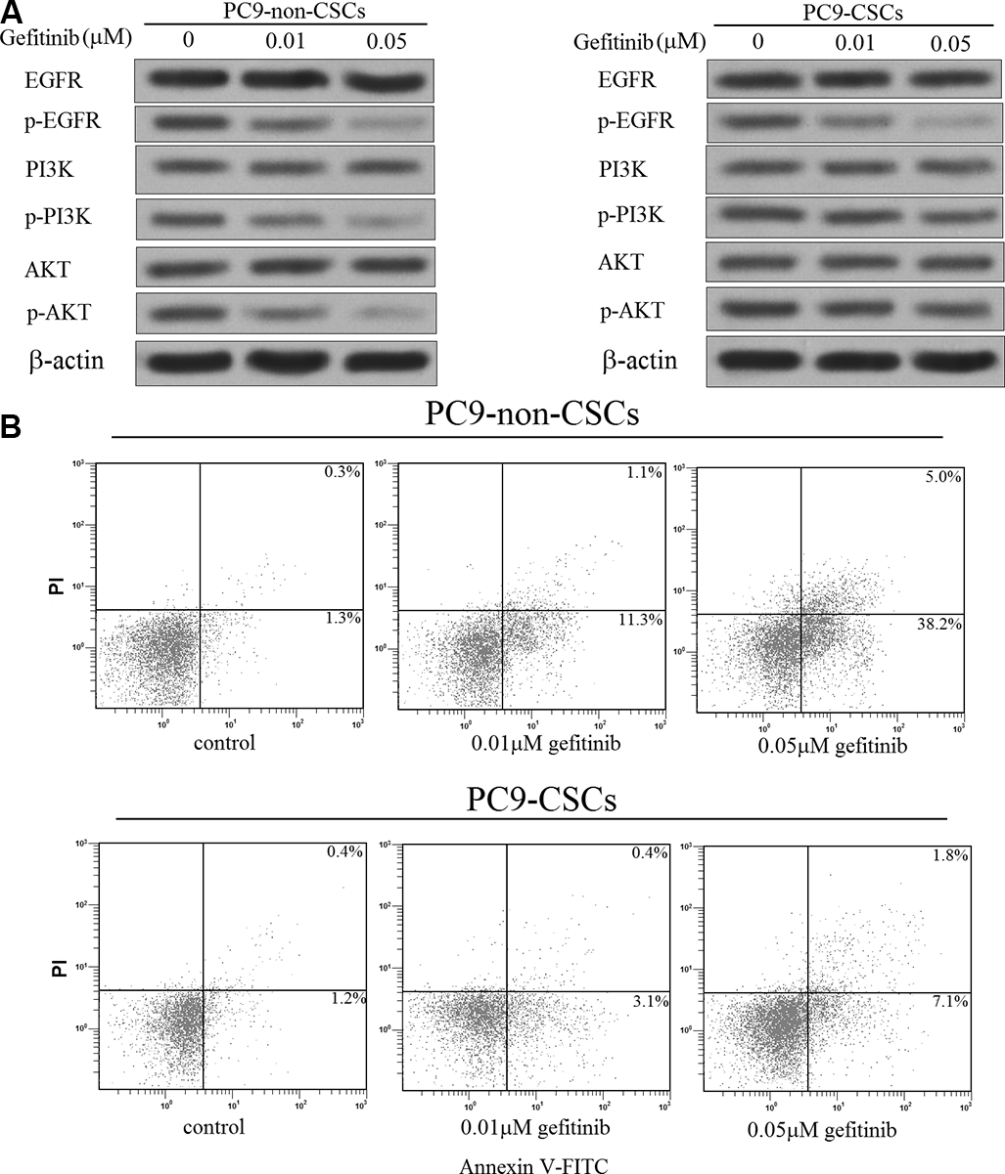
Gefitinib failed to induce the PI3K/AKT-related apoptosis obviously in the PC9-CSCs (**A**) After treatment with 0.1 μM or 0.5 μM gefitinib, the phosphorylation of EGFR, PI3K and AKT was evaluated by western blot analysis in the PC9-CSCs and PC9-non-CSCs. (**B**) After treatment with 0.1 μM or 0.5 μM gefitinib, the apoptosis of PC9-CSCs and PC9-non-CSCs was detected by flow cytometry.

As the preceding results have shown that the PC9-CSCs are resistant to gefitinib, we next investigate the role of gefitinib in the EGFR/PI3K/AKT signaling pathway. As shown in Figure 2A, we found that the entire EGFR/PI3K/AKT signaling pathway was inhibited by the gefitinib treatment in the PC9-non-CSCs. However, interestingly, we observed that although the gefitinib treatment significantly inhibited the phosphorylation of EGFR, it failed to suppress the activation of PI3K/AKT pathway in the PC9-CSCs. Previous researches have demonstrated that the apoptosis of cancer cells is inhibited by the PI3K/AKT pathway [21, 22]. We therefore investigated the effect of gefitinib on inducing the apoptosis in the PC9 cells. In accordance with the results shown in Figure 2A, we found that the gefitinib significantly induced the apoptosis in the PC9-non-CSCs, whereas it failed to trigger the apoptosis obviously in the PC9-CSCs (Figure 2B).

### Overexpression of miR-128 increases the sensitivity of PC9-CSCs to gefitinib treatment *in vitro*

**Figure 3:**
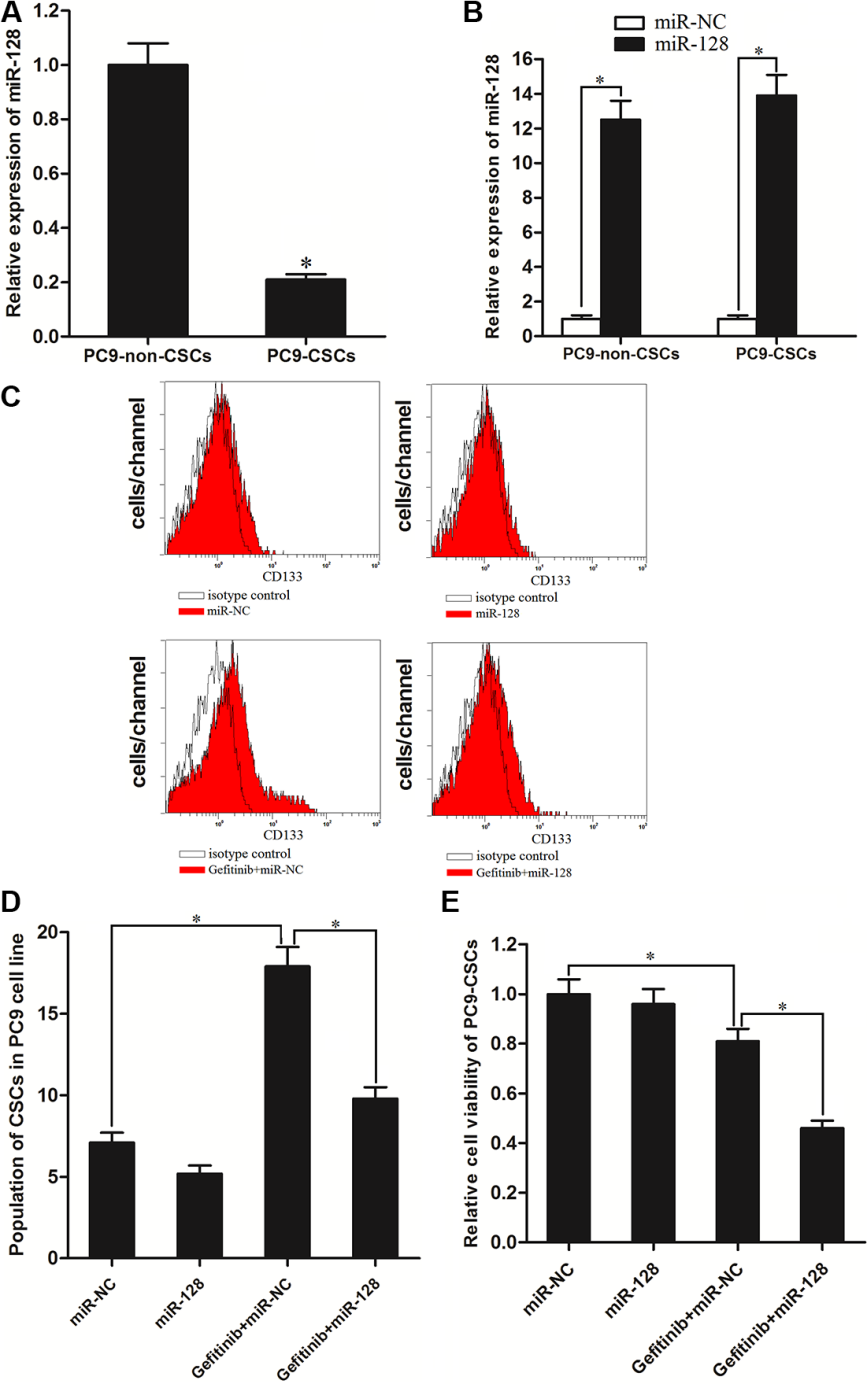
MiR-128 enhanced the cytotoxicity of gefitinib to PC9-CSCs (**A**) QRT-PCR analysis showed that the expression of miR-128 was significantly decreased in the PC9-CSCs compared with the PC9-non-CSCs. (**B**) Transfection of miR-128 mimics increased the levels of miR-128 in the PC9-CSCs and PC9-non-CSCs. (**C**) The population of CD133^+^ CSCs was determined by FACS in PC9 cells. (**D**) The effect of gefitinib and miR-128 on changing the CSCs population in PC9 cells. (**E**) After the PC9-CSCs were treated with miR-128 mimics and gefitinib (0.05 μM). The relative cell viability was determined by MTT assay. *P < 0.05.

To investigate the role of miR-128 in gefitinib treatment *in vitro*, the expression of miR-128 between PC9-CSCs and PC9-non-CSCs was compared by qRT-PCR. The results of qRT-PCR revealed that the miR-128 expression level was significantly decreased in the CSCs compared with the non-CSCs in the PC9 cell line (Figure 3A). To study the role of miR-128 in PC9-CSCs, we transfected these cells with miR-128 mimics. As shown in Figure 3B, transfection of miR-128 significantly increased the expression level of miR-128 in the PC9-CSCs as well as the corresponding non-CSCs. Interestingly, we found the treatment of gefitinib significantly increased the population of CD133 positive CSCs in PC9. By contrast, although the transfection of miR-128 didn't decrease the percentage of PC9-CSCs population significantly, it inhibited the effect of gefitinib on enriching the PC9-CSCs population (Figure 3C and 3D). We explain that the CSCs could survive under the gefitinib treatment, whereas the overexpression of miR-128 increased the sensitivity of PC9-CSCs to gefitinib. To confirm the effect of miR-128 on the gefitinib treatment to PC9-CSCs, we next performed a cell viability assay. As shown in Figure 3E, although the single treatment of miR-128 didn't show significant cytotoxicity, it significantly sensitized the PC9-CSCs to gefitinib-induced cell death. Taken together, these results prove that the overexpression of miR-128 is able to increase the sensitivity of PC9-CSCs to gefitinib treatment *in vitro*.

### Overexpression of miR-128 increases the anti-tumor effect of gefitinib on NSCLC *in vivo*

**Figure 4:**
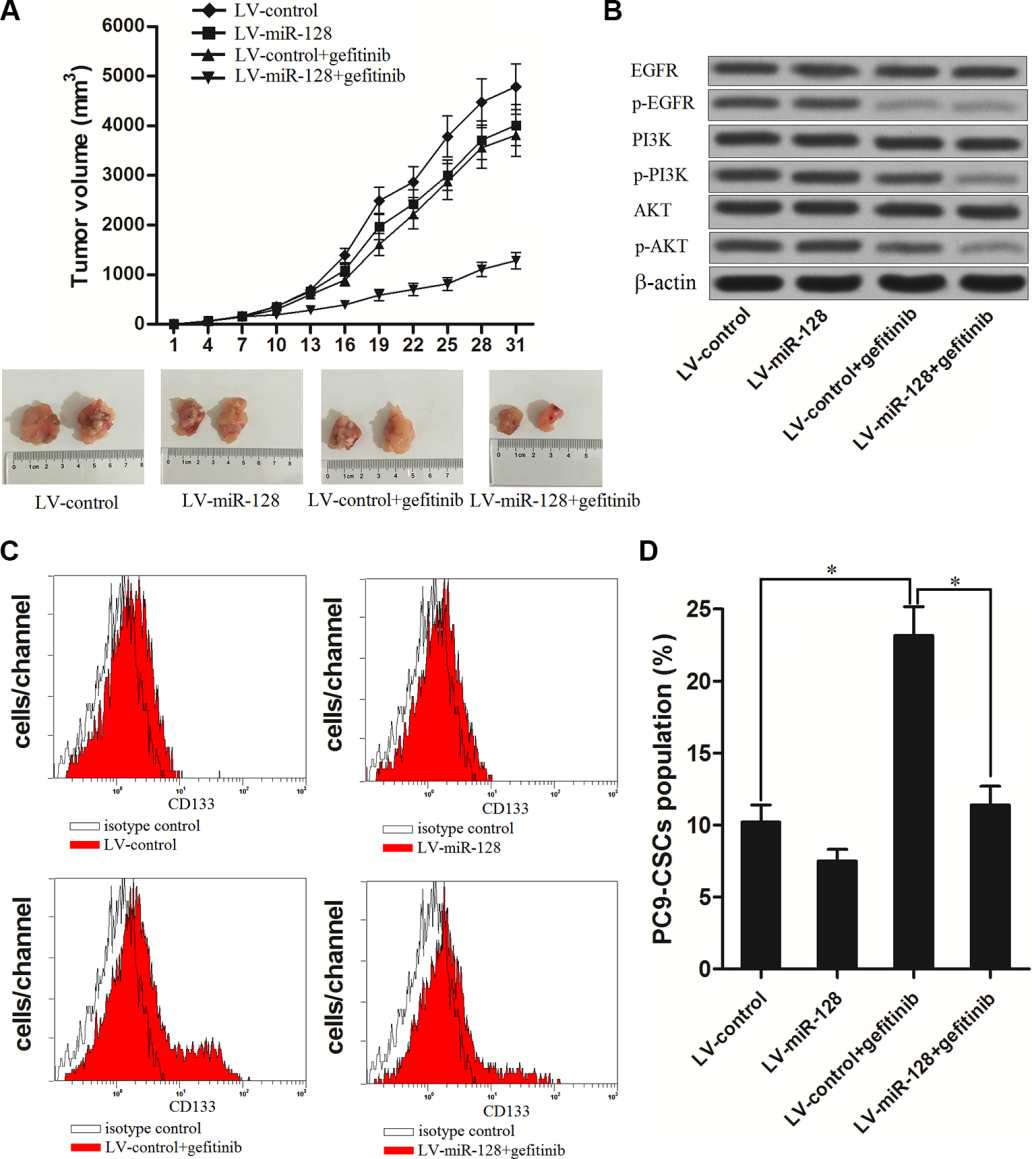
Enforced expression of miR-128 enhanced the anti-tumor effect of gefitinib on NSCLC *in vivo* (**A**) Volumes of PC9 originated tumors *in vivo* were detected every three days with or without miR-128 transfection and gefitinib treatment. (**B**) The phosphorylation of EGFR, PI3K and AKT was evaluated by western blot analysis in the tumor tissues originated from the LV-miR-128 transfected PC9 or LV-control transfected PC9 under the treatment of gefitinib. (**C**) The population of CSCs in tumor tissue cells *in vivo* was detected by flow cytometry. (**D**) overexpression of miR-128 inhibited the effect of gefitinib on enriching the population of CSCs *in vivo*. *P < 0.05.

To verify whether miR-128 promotes the anti-tumor effect of gefitinib on NSCLC *in vivo*, we inoculated the nude mice with the PC9 cells transfected with LV-miR-128 or LV-control. The results revealed that miR-128 could promote the gefitinib to decrease the PC9 growth *in vivo* (Figure 4A). In the tumor tissues originated from the LV-miR-128 transfected PC9, we found that the gefitinib treatment significantly inhibited the phosphorylation of the entire EGFR/PI3K/AKT signaling pathway. However, in the tumor tissues originated from the LV-control transfected PC9, although the activation of EGFR was obviously suppressed, the phosphorylation of PI3K and AKT was inhibited only slightly under the treatment of gefitinib (Figure 4B). Furthermore, in the LV-control transfected PC9 tumor tissues, the treatment of gefitinib resulted in significant up-regulation of PC9-CSCs population. On the contrary, overexpression of miR-128 could inhibit the enrichment of CSCs population due to the gefitinib therapy (Figure 4C and 4D). These results suggest that the failure of gefitinib treatment may due to the drug-resistance of lung cancer stem cells, and the enforced expression of miR-128 is potential strategy to increase the sensitivity of lung cancer stem cells to gefitinib.

### C-met is the target of miR-128 in PC9

**Figure 5:**
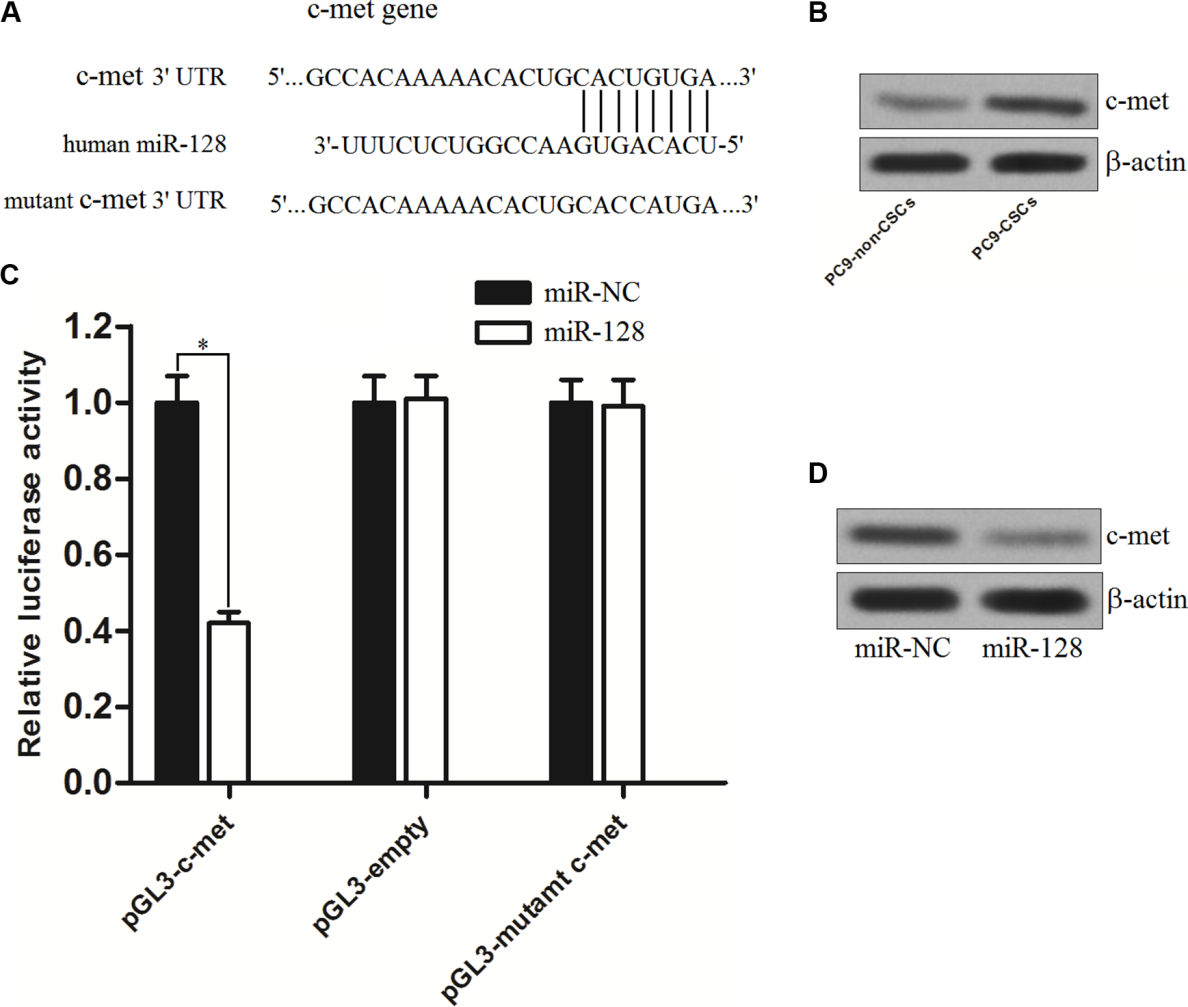
C-met is the target of miR-128 in PC9 (**A**) C-met is predicted as the target of miR-128 by the TargetScan, miRanda, and PicTar databases. (**B**) The expression of c-met in PC9-non-CSCs and PC9-CSCs was evaluated by western blot analysis. (**C**) PC9-CSCs were co-transfected with wildtype/mutant 3′-UTR of c-met and miR-128 mimics as indicated. 48 h post transfection, luciferase activity was detected using Dual-Luciferase Reporter Assay System according to the manufacturer's instruction. (**D**) The effect of miR-128 on regulating the protein level of c-met in PC9-CSCs was evaluated by western blot analysis. *P < 0.05.

To understand how miR-128 facilitates gefitinib-induced cell death in PC9-CSCs, the TargetScan, miRanda, and PicTar databases were used to predict the targets of miR-128. Of these target genes that were predicted by these databases, the c-met gene is considered as the possible target because it was commonly predicted by all of these databases and owned a highly conserved sequence in the 3′ UTR of the c-met mRNA that was targeted by miR-128 (Figure 5A). To investigate the potential negative correlation between miR-128 and c-met, we evaluated the expression of c-met in the PC9-CSCs and PC9-non-CSCs. As shown in Figure 5B, we observed that the expression level of c-met was significantly higher in the PC9-CSCs than that in the PC9-non-CSCs. We then cloned the c-met 3′-UTR sequences containing the predicted target site of miR-128 into a luciferase reporter vector. The results of luciferase reporter assays showed that the luciferase activity in the group co-transfected with miR-128 and pGL3-c-met reporter was significantly lower than that in the group with miR-NC (Figure 5C), implying the effect of miR-128 on inhibiting the mRNA of c-met gene. Furthermore, the results of western blot analysis demonstrated that the overexpression of miR-128 could decrease the protein level of c-met in the PC9-CSCs (Figure 5D). Taken together, we prove that the c-met gene is a functional target of miR-128 in PC9-CSCs.

### MiR-128 increases the sensitivity of PC9-CSCs to gefitinib via down-regulating the expression of c-met

**Figure 6:**
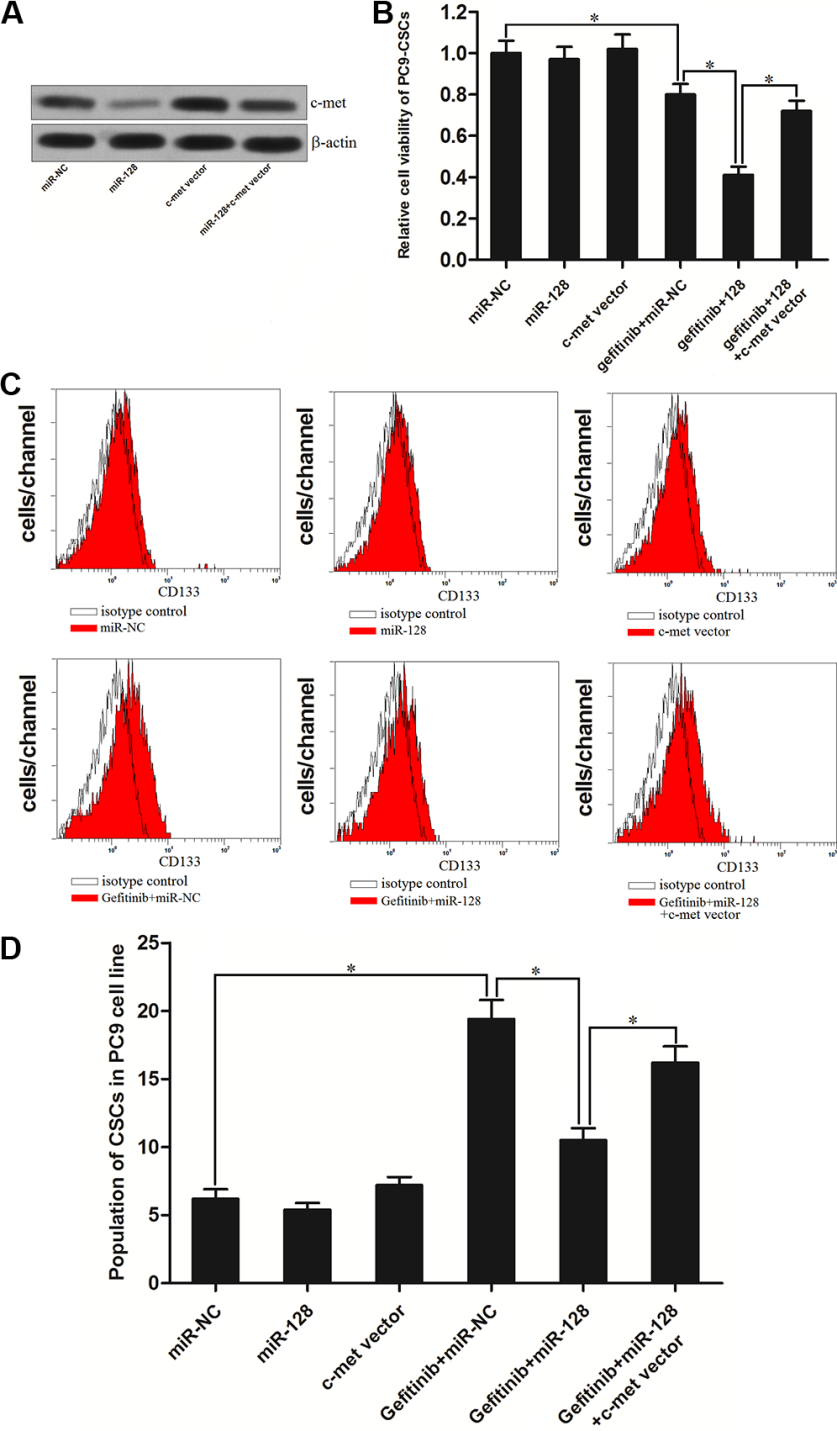
MiR-128 promoted the gefitinib-induced cell death via down-regulating the expression of c-met in PC9-CSCs (**A**) Transfection of c-met recombinant eukaryotic expression vector abolished the inhibition of c-met induced by the miR-128 in PC9-CSCs. (**B**) C-met vector abolished the MiR-128 promoted cell death induced by the gefitinib (0.05 μM) in the PC9-CSCs. (**C**) The population of CSCs in PC9 cells was detected by flow cytometry after they were treated with miR-128, gefitinib (0.05 μM), and c-met vector. (**D**) c-met vector abolished the effect of miR-128 on inhibiting the gefitinib-induced enrichment of CSCs population in PC9 cells. *P < 0.05.

To study the role of c-met in the miR-128 promoted cell death in PC9-CSCs, we increased the expression of c-met by its recombinant eukaryotic expression vector to “rescue” the miR-128 promoted cell death. We found the transfection of c-met vector abolished the effect of miR-128 on suppressing the expression of c-met (Figure 6A). As expected, we observed that although the miR-128 significantly enhanced the cytotoxicity of gefitinib to PC9-CSCs, the enforced expression of c-met by its recombinant plasmid abolished the promotion of miR-128 on the gefitinib-induced cell death (Figure 6B). Furthermore, transfection of c-met plasmid also abolished the effect of miR-128 on inhibiting the gefitinib-induced enrichment of CSCs population in PC9 cells (Figure 6C and 6D). Taken together, these results indicate that the miR-128 is able to increase the sensitivity of PC9-CSCs to gefitinib by down-regulating the expression of c-met.

### MiR-128 enhanced the gefitinib-induced apoptosis by suppressing the PI3K/Akt pathway

**Figure 7:**
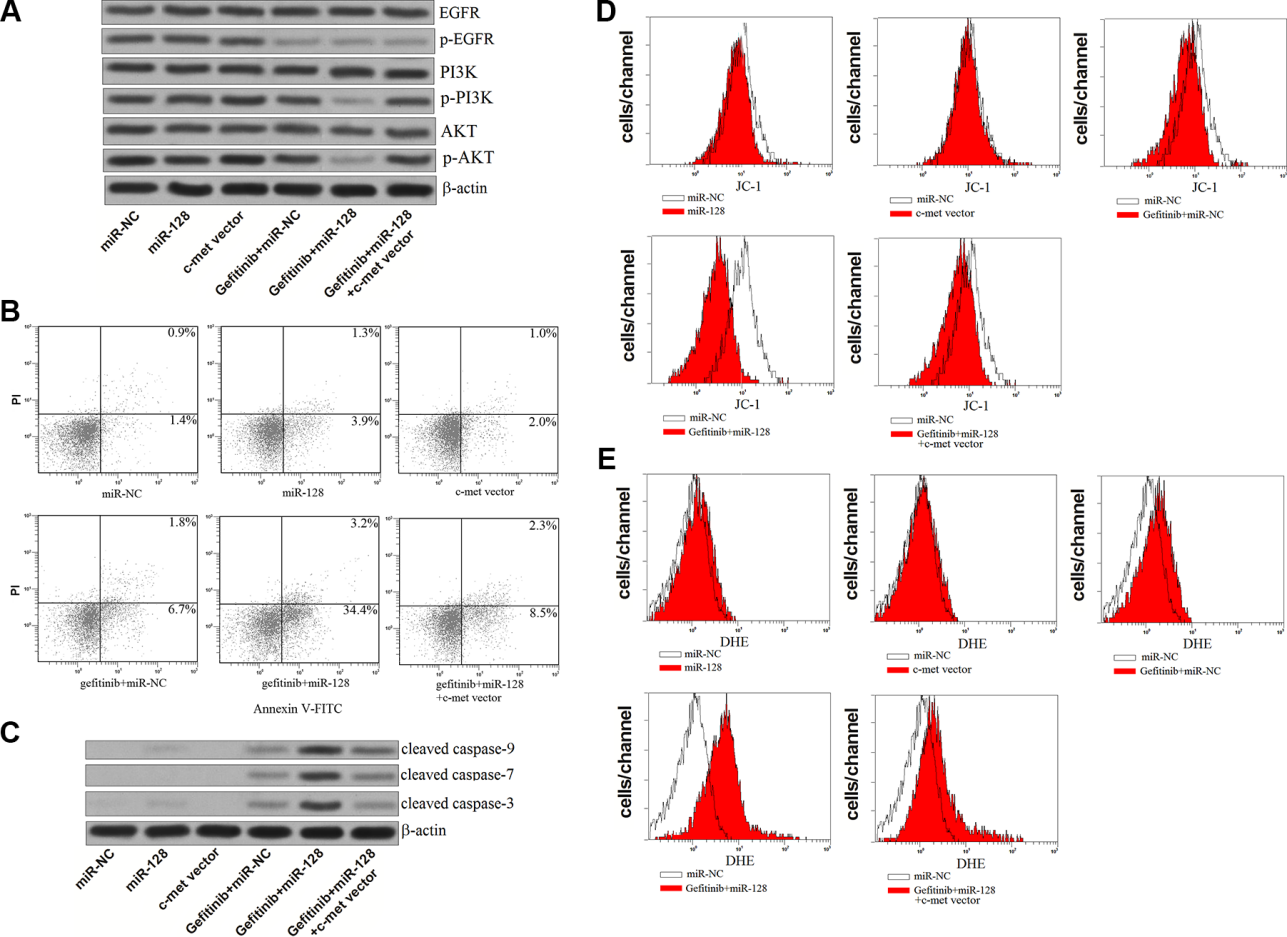
MiR-128 enhanced the gefitinib-induced apoptosis by suppressing the PI3K/Akt pathway (**A**) Western blot analysis was performed to evaluate the phosphorylation of EGFR, PI3K and Akt in the PC9-CSCs treated with miR-128, gefitinib (0.5 μM) and c-met vector. (**B**) Flow cytometry was performed to detect the apoptosis of PC9-CSCs treated with miR-128, gefitinib (0.5 μM) and c-met vector. (**C**) Western blot analysis was performed to evaluate the cleavage of caspase-9, caspase-7 and caspase-3 in the PC9-CSCs treated with miR-128, gefitinib (0.5 μM) and c-met vector. (**D**) The MMP of PC9-CSCs treated with miR-128, gefitinib (0.5 μM) and c-met vector was measured by JC-1 staining on the flow cytometry. (**E**) The generation of ROS in PC9-CSCs treated with miR-128, gefitinib (0.5 μM) and c-met vector was measured by DHE staining on the flow cytometry.

Previous studies have demonstrated that c-met amplification leads to the resistance of EGFR-TKIs by activating the downstream of PI3K/AKT signaling pathway [23, 24]. We therefore investigated the role of miR-128/c-met axis in the apoptosis pathway in the PC9-CSCs treated with gefitinib. As shown in Figure 7A, single treatment of gefitinib only inhibited the phosphorylation of EGFR without influencing the activation of PI3K and AKT obviously, whereas the combination with gefitinib and miR-128 significantly suppressed the entire EGFR/PI3K/AKT pathway. However, the overexpression of c-met “recovered” the activation of PI3K/AKT even the PC9-CSCs were co-treated with gefitinib and miR-128. As the results of PI3K/AKT inhibition, we observed that the overexpression of miR-128 significantly promoted the gefitinib-induced apoptosis, meanwhile the synergistic effects of miR-128 was abolished by the c-met vector (Figure 7B). To investigate the apoptosis pathway induced by the gefitinib and miR-128, the caspases and mitochondria were studied. As we observed, transfection of miR-128 significantly promoted the activation of caspase-9, −7, −3 induced by gefitinib in the PC9-CSCs (Figure 7C). Furthermore, we found the mitochondrial membrane potential (MMP) of PC9-CSCs was significantly decreased due to the co-treatment with gefitinib and miR-128 (Figure 7D). As the results of MMP collapse, the ROS which is considered as a key apoptotic inducer [25] was released from the mitochondria into the cytoplasm (Figure 7E). In addition, enforced expression of c-met obviously abolished the effect of the combination with gefitinib and miR-128 on both the casspases and mitochondria. Taken together, we demonstrate that the miR-128/c-met axis inhibited the activation of PI3K/AKT and the downstream of mitochondrial apoptosis pathway in the gefitinib-treated PC9-CSCs.

## DISCUSSION

Increasing evidence has indicated that miRNAs are related with the drug-resistance of the tumor cells to cancer therapy [26–28]. Although these researches have demonstrated the responsibility of miRNAs dysregulation for the drug-resistance, the role of miRNAs in regulating the drug-resistance of CSCs is not clear. Reports have indicated that miR-128 acts as a tumor suppressor in several cancers. In these studies, miR-128 inhibits cell proliferation, migration and invasion of the cancer [29, 30].

In the present study, we found that the expression of miR-128 was decreased in the lung cancer stem cells. Furthermore, we demonstrated that the down-regulation of miR-128 was associated with the gefitinib resistance in these cells. Overexpression of miR-128 was then found to increase the sensitivity of PC9-CSCs to gefitinib and therefore inhibited the effect of gefitinib on enriching the CSCs population *in vitro* and *in vivo*. Thus, for the first time, we reported that miR-128 can reverse the resistance and enhance the anti-tumor effect of gefitinib on NSCLC by decreasing the CSCs population.

EGFR amplification or mutation occurs in multiple cancers, especially NSCLC. These EGFR mutated lung cancer cells (such as PC9) are sensitive to the EGFR-TKIs including gefitinib. Therefore the NSCLC patients could benefit from the gefitinib treatment [31, 32]. In the EGFR pathway, the activation of EGFR leads to the phosphorylation of PI3K and the subsequent generation of phosphatidylinositol-3,4,5-trisphosphate (PIP3), which in turn triggers the AKT. The activated AKT can promote cell growth and inhibit apoptosis. Thus, the PI3K/AKT pathway contributes to the tumor development [33–35]. Interestingly, according to the results of this study, we found the gefitinib failed to suppress the activation of PI3K/AKT pathway in the lung cancer stem cells *in vitro* and *in vivo*, whereas the EGFR signaling could be inhibited by this kind of EGFR-TKI. We therefore demonstrated that the activation of PI3K/AKT pathway was independent on the EGFR signaling in the lung cancer stem cells.

C-met is the receptor for hepatocyte growth factor (HGF). The HGF/c-met signaling was reported to promote the tumorigenicity in a variety of manners including the highly oncogenic PI3K/AKT pathway [36, 37]. Researches have indicated that inhibition of c-met and its downstream signaling could be the potential strategy to enhance the therapeutic efficacy of cancer [38]. Furthermore, the activation of c-met pathway was identified as an important mechanism of acquired resistance to gefitinib [39]. Recently, it's reported that c-met is inhibited by miRNAs in lung cancer, and the miRNAs/c-met axis has been proved to be associated with the gefitinib resistance [40].

**Figure 8:**
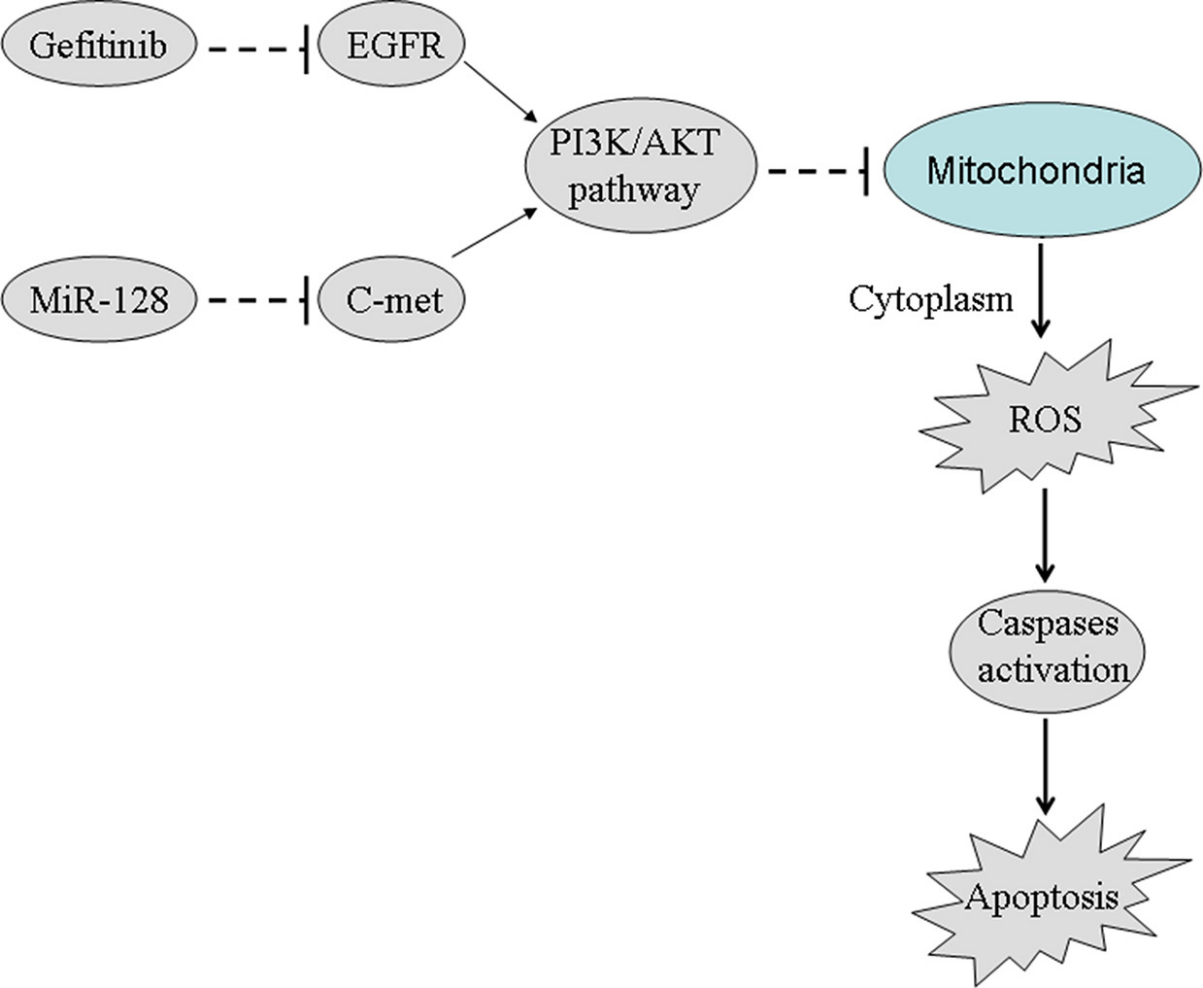
Schema of the predicted mechanisms implicated in PC9-CSCs response to gefitinib and miR-128 Combination with miR-128 and gefitinib inhibits the PI3K/AKT pathway and induces the cell apoptosis by suppressing the expression of c-met in PC9-CSCs.

In this study, we demonstrated that the gene of c-met was overexpressed in the lung cancer stem cells. We also proved that the miR-128/c-met axis determined the sensitivity of the lung cancer stem cells gefitinib *in vitro* and *in vivo*. Furthermore, combination with gefitinib and miR-128 inhibited the PI3K/AKT pathway. Subsequently, the PC9–CSCs underwent the collapse of mitochondria, release of ROS, cleavage of caspases, and finally, the apoptosis occurred. These results provide several pieces of evidence to prove that overexpression of miR-128 is able to reverse the gefitinib-resistant lung cancer stem cells by inhibiting the c-met/PI3K/AKT pathway (Figure 8). Therefore, combination with miR-128 and EGFR-TKIs could be attractive treatments for patients with NSCLC.

## MATERIALS AND METHODS

### Animals

Four-week-old female nude mice (BALB/c, nu/nu; 17-22 g in weight) were purchased from the Shanghai Laboratory Animal Center (Shanghai, China) and kept in a room under a 12 h artificial light/dark cycle with free access to food and water. Animal care and the experimental procedures in this study were approved by the Animal Care Committee of First Affiliated Hospital, School of Medicine, Zhejiang University and complied with the recommendations of the Chinese guidelines for the care and use of laboratory animals.

### Cell culture

The NSCLC cell line PC9 was purchased from American Type Culture Collection (USA). For sorting the PC9-CSCs, the PC9 cells were stained with CD133-FITC antibody (Miltenyi Biotec, Germany) for 20 min at room temperature. Then the CD133^+^ PC9 cells were sorted as the PC9-CSCs on a FACS vantage (FACSCALIBUR, BD Biosciences, USA). In addition, the CD133^−^ PC9 cells were sorted and considered as the PC9-non-CSCs. Cells were cultured in DMEM basic medium (Gibco, USA) contained with 10% fetal bovine serum (Gibco) at 37°C in a humidified 5% CO2 incubator. To evaluate the role of miR-128 *in vivo*, the stable PC9 cell line overexpressed miR-128 was generated. Briefly, we purchased the recombinant lentivirus which contains miR-128 precusor sequence from the Shanghai Genechem Co., Ltd. (Shanghai, China). The precusor sequence of miR-128 is as follows: 5′-UGAGCUGUUGGAUUCGGGGC CGUAGCACUGUCUGAGAGGUUUACAUUUCUCACAGUGAACCGGUCUCUUUUUCAGCUGCUUC-3′. Then, 1 × 10^4^ PC9 cells were transfected with 5 × 10^5^ transducing units of lentivirus, and the cells were selected with 1 μg/ml puromycin for 2 weeks.

### Quantitative reverse transcriptase real time PCR (qRT-PCR)

RNA was extracted from cells using the TRIzol reagent (Invitrogen, USA). The reverse transcription of miR-128 is performed by using Hairpinit^™^ miR qPCR quantification kit (GenePharma, China). Then, the real time PCR was performed in triplicate using the SYBR Premix Ex Taq (TaKaRa, Japan) according to the manufacturer's instructions. The expression of miR-128 was determined using the 2^−^^ΔΔCT^ analysis method [41] taking the U6 snRNA as the internal control.

### Transfection

The c-met expression vector was conducted by cloning the open reading frame of c-met gene into the pcDNA3.1 plasmid (Life Technologies, USA). The miR-128 mimics and negative control (miR-NC) were synthesized by RiboBio Co. Ltd. (China). The sequence of miR-128 and miR-NC are as follows: 5′-UCACAGUGAACCGGUCUCUUU-3′ (miR-128), 5′-UGUCCUCCU GGAAUUACACGU-3′ (miR-NC). For transfection, cells were seeded and transfected with 2 mg/ml c-met vector, 50 pmol/ml miR-128 mimics or miR-NC using Lipofectamine 2000 (Invitrogen) according to the manufacturer's guidance.

### Cell viability assay

A total of 5 × 10^3^ cells were seeded into 96-well plates and grown for 24 h post-transfection. Then, cells were incubated in gefitinib for another 48 h. Subsequently, 20 ml 3-(4, 5-dimethylthiazol-2-yl)-2, 5-diphenyltetrazolium bromide (MTT) (5 mg/ml, Sigma-Aldrich, USA) was added and incubated at 37°C for 4 hours. Following incubation, the media was carefully removed and 150 μl of DMSO was added at room temperature for 30 min. The absorbance in each well was measured at 570 nm using a microplate reader (Sunrise Microplate Reader, TECAN, Switzerland). In addition, the half maximal inhibitory concentration (IC50) of gefitinib to the PC9-CSCs and PC9-non-CSCs was calculated according to the viability curves.

### Western blot analysis

After treatment, proteins were isolated with RIPA buffer (Cell Signaling Technology, USA), and then quantified using the Bradford procedure (Bio-Rad, USA). Approximately 50 μg of total proteins were then separated by 12.5% sodium dodecyl sulfate polyacrylamide gel electrophoresis (SDS-PAGE) and transferred to a PVDF membrane (Millipore, USA). Membranes were incubated in 5% skim milk for 1 h at room temperature and then incubated with the primary antibodies (anti-EGFR, anti-phosphorylated EGFR, anti-PI3K, anti-phosphorylated PI3K, anti-AKT, anti-phosphorylated AKT, anti-c-met, anti-cleaved caspase-9, anti-cleaved caspase-7, anti-cleaved caspase-3 and anti-β-actin, all of them purchased from Cell Signaling Technology) overnight at 4°C. A fluorescent goat anti-rabbit (Cell Signaling Technology) secondary antibody was used and the protein bands were then detected with an enhanced chemilu-minescence detection kit (Pierce, USA). The level of β-actin was used to normalize the protein levels.

### Apoptosis analysis

After treatment, cells were stained with PI and Annexin-V (Sigma-Aldrich, USA) for 20 min at room temperature according to the manufacturer's guidance. The percentage of apoptotic cells was quantified using the flow cytometry.

### Luciferase reporter assay

To construct the c-met 3′UTR reporter plasmid, the putative binding sites of miR-128 in the 3′UTR of the human c-met gene was amplified and inserted downstream of the luciferase gene in the luciferase reporter pGL3 Luciferase Reporter Vectors (Promega, USA) and named pGL3-c-met. The mutant c-met reporter was created by mutating the seed regions of the miR-128 binding sites (CACUGUGA) by using the site-directed mutagenesis kit (Takara, Japan) and named pGL3-mutant c-met. For luciferase reporter assay, cells were seeded and co-transfected with 50 pmol/ml miR-128 mimics together with 2 mg/ml Firefly luciferase reporters and 100 ng/ml Renilla luciferase pRL-TK vector (Promega). Firefly and Renilla luciferase activities were measured consecutively using the Dual-Luciferase Reporter assay system (Promega) according to the manufacturer's instructions.

### Detection of mitochondrial membrane potential (MMP, ΔΨm) and reactive oxygen species (ROS)

After treatment, the MMP was detected using the 5,5′,6,6′-Tetrachloro-1,1′,3,3′-tetraethyl imidacarbo cyanine iodide (JC-1, Molecular Probes, USA) as the indicator according to the manufacturer's instructions. For measurement of ROS, the dihydroethidium (DHE, Molecular Probes) was used according to the manufacturer's instructions. Both of the MMP and ROS were analyzed on the flow cytometry.

### Tumor growth in nude mice

An equal number (5 × 10^6^) of PC9 cells transfected with lentivirus-miR-128 (LV-miR-128) or lentivirus-control (LV-control) were harvested and washed. The experimental mice were randomized into four groups (8 mice/group). For the tumorigenesis assay, two groups of mice were subcutaneously injected with LV-control PC9 cells (for LV-control group and LV-control + gefitinib group, respectively), and two groups of mice were subcutaneously injected with LV-miR-128 PC9 cells (for LV-miR-128 group and LV-miR-128 + gefitinib group, respectively). The tumor volume (V) was calculated according to the formula: length × (width^2^)/2. In LV-control + gefitinib group and LV-miR-128 + gefitinib group, the mice began to receive the gefitinib treatment (30 mg/kg/d, via gavage for two weeks) through oral administration once daily when the tumors reached a mean volume of 100 mm^3^ (42). The mice were monitored every three days for tumor formation using the calipers. Nude mice were euthanized at the experimental end-point (31 days post-injection).

For detection of CSCs in tumor tissues, cells were purified as described previously using the collagenase type III (43). The population of CSCs was analyzed using CD133-FITC antibody on the FACS vantage.

### Statistical analysis

All of the experiments were repeated in triplicate and the experimental data were expressed as the mean ± SD. For comparison analysis, the statistical analysis was performed by student's *t-test* using SPSS 16.0 software. Values of *P* < 0.05 were considered significant.
